# HZ-A-018, a novel inhibitor of Bruton tyrosine kinase, exerts anti-cancer activity and sensitizes 5-FU in gastric cancer cells

**DOI:** 10.3389/fphar.2023.1142127

**Published:** 2023-03-22

**Authors:** Danjing Liu, Wei Xu, Bin Lin, Cong Ji, Minmin Shen, Shuying Shen, Junjie Ma, Xinglu Zhou, Youyou Yan, Bo Zhang, Nengming Lin

**Affiliations:** ^1^ College of Pharmaceutical Sciences, Hangzhou First People’s Hospital, Zhejiang Chinese Medical University, Hangzhou, Zhejiang, China; ^2^ Key Laboratory of Clinical Cancer Pharmacology and Toxicology Research of Zhejiang Province, Affiliated Hangzhou First People’s Hospital, Zhejiang University School of Medicine, Huzhou, Zhejiang, China; ^3^ Key Laboratory of Intelligent Pharmacy and Individualized Therapy of Huzhou, Changxing People’s Hospital, Huzhou, Zhejiang, China; ^4^ Huzhou Central Hospital, Affiliated Huzhou Hospital, Zhejiang University School of Medicine, Huzhou, Zhejiang, China; ^5^ Hangzhou Hezheng Pharmaceutical Co., Ltd., Huzhou, Zhejiang, China; ^6^ Cancer Center, Zhejiang University, Huzhou, Zhejiang, China; ^7^ Westlake Laboratory of Life Sciences and Biomedicine of Zhejiang Province, Huzhou, Zhejiang, China

**Keywords:** 5-FU, Bruton tyrosine kinase, Akt, RRM2, gastric cancer

## Abstract

Gastric cancer is the third leading cause of cancer related death worldwide. Due to the complexity and heterogeneity of gastric cancer, the development of targeted drugs is somehow limited, but is urgently needed. Since the expression of Bruton tyrosine kinase (BTK) was significantly associated with the prognosis of gastric cancer patients, we aimed to determine the anti-cancer activity of HZ-A-018, which was a novel derivative of ACP-196, in gastric cancer cells. As a result, HZ-A-018 presented a stronger anti-proliferation activity than ACP-196 *via* the substantial suppression of AKT/S6 pathway. In addition, HZ-A-018, but not ACP-196, exerted the synergistic effects in combined treatment with 5-FU both *in vitro* and *in vivo*, without exacerbating the adverse effects of 5-FU. Mechanismly, the combination of HZ-A-018 and 5-FU remarkably reduced the expression of RRM2, which played an essential role in proliferation and drug sensitivity in gastric cancer cells. In summary, our work demonstrated the stronger anti-cancer activity of HZ-A-018 than ACP-196 in gastric cancer cells, and revealed synergistic effects of HZ-A-018 and 5-FU combination probably through the inhibition of RRM2 *via* AKT/S6 pathway, thereby providing a promising therapeutic strategy in gastric cancer.

## Introduction

Gastric cancer is the fourth most common cancer worldwide (Sung et al., 2021). Due to its malignant status and molecular diversity, gastric cancer accounts for over one million of newly diagnosed cases and approximately 700,000 deaths in 2021 (Wang et al., 2022a). Currently, 5-fluorouracil (5-FU)-based chemotherapy is the most commonly used therapeutic strategy for gastric cancer patients after surgical resection (Kim et al., 2019), (Bang et al., 2019), whereas the efficacy of chemotherapy is somehow unsatisfied especially in replapsed patients, attributed to the complexity of gastric cancer (Joshi and Badgwell, 2021), (Yeoh and Tan, 2022). In addition, the heterogeneity of gastric cancer substantially increase the difficulty of developing targeted drugs, rendering the lack of targeting agents for gastric cancer in clinic (Garcia-Pelaez et al., 2021), (Wang et al., 2022b). Therefore, the exploration of novel targets in gastric cancer is desperately in need.

Bruton’s tyrosine kinase (BTK) is known to regulate multiple anti-apoptotic pathways, including PI3K-AKT, STAT5 and NF-kB, in B-lineage lymphoid cells (Hu et al., 2021), (Purvis et al., 2020), and has merged as an efficacious therapeutic target for the treatment of leukemia. For instance, the second-generation BTK inhibitor Acalabrutinib (also known as ACP-196) has been approved by FDA for the treatment of adults with chronic lymphocytic leukemia (Wang et al., 2018), (Roskoski, 2019). Recently, an aberrant overexpression of BTK is also found in breast cancer, ovarian cancer and colorectal cancer (Lavitrano et al., 2020), (Molina-Cerrillo et al., 2017), and the suppression of BTK by specific inhibitors or RNA interference has shown an enhanced chemosensitivity of cancer cells and the substantial inhibition of tumor growth (Yeh et al., 2021), (Chen et al., 2021a). However, the efficacy of BTK inhibitors in gastric cancer remains unclear. HZ-A-018 was a novel derivative of ACP-196, and was currently under an ongoing clinical trial (NCT04173455) to treat B Cell lymphoma. In this study, we aimed to determine the anti-cancer effects of HZ-A-018, and explored its underlying mechanisms.

5-fluorouracil (5-FU) is the most commonly used chemotherapy drug to treat gastric cancer, and it works by inhibiting essential biosynthetic processes or being incorporated into DNA or RNA (Vodenkova et al., 2020), (Longley et al., 2003). In an early work, it was found that the clinical response to 5-FU was significantly associated with the transcript level of ribonucleotide reductase subunit M2 (RRM2) in colorectal carcinoma (Kunicka et al., 2016), (Kidd et al., 2005). In recent studies, RRM2 was reported to control tumor progression and drug resistance (Gandhi et al., 2020), and was able to regulate the sensitivity of renal cancer cells to Sunitinib and PD-1 Blockade *via* modulating AKT pathway (Xiong et al., 2021). However, a direct evidence that demonstrated the correlation between RRM2 expression and 5-FU sensitivity in gastric cancer is still lacking. Meanwhile, considering the inhibitory effects of HZ-A-018 on AKT/S6 pathway, the influence of HZ-A-018 on RRM2 was also evaluated in this study.

Herein, we found that HZ-A-018 displayed a stronger anti-proliferation efficacy than ACP-196 through the suppression of AKT/S6 signaling pathway in two gastric cancer cell lines. Moreover, the combination treatment of HZ-A-018 and 5-FU displayed a synergistic effect both *in vitro* and *in vivo*, thus providing a promising therapeutic strategy in the treatment for gastric cancer.

## Materials and methods

### Cell lines

Human gastric cancer cell lines HGC-27 and BGC-823 were purchased from the Chinese Academy of Sciences (Shanghai, China). Cells were cultured in RPMI-1640 containing 10% fetal bovine serum and 100 U ml‒1penicillin/streptomycin at 37°C in 5% CO_2_ in a humidified atmosphere. HZ-A-018, ACP-196 and 5-fluorouracil were dissolved in DMSO at a concentration of 10 mM respectively.

### Antibodies and reagents

5-FU, ACP-196 and MK-2206 were purchased from Selleck. HZ-A-018 was provided by Hangzhou Hezheng Pharmaceutical Co. Ltd. RPMI-1640 medium and fetal bovine serum (FBS) were purchased from Gibco (Grand Island, NY, United States). The Annexin V-FITC Apoptosis Kit was purchased from BestBio (Shanghai, China). The primary antibodies against poly (ADP-ribose) polymerase (PARP), cleaved PARP, procaspase-3, cleaved caspase-3, PI3 Kinase p110α, phospho-mTOR (Ser2448), mTOR, phospho-AKT (Ser 473), AKT, Phospho-p70 S6 Kinase (Thr421/Ser424), p70 S6 Kinase, phospho-S6 Ribosomal Protein (Ser240/244), phospho-S6 Ribosomal Protein (Ser235/236), S6 Ribosomal Protein, RRM2, E2F1 were purchased from Cell Signaling Technology (Beverly, MA, United States). The primary antibodies against BTK (phospho Y223), Mcl-1, Bim and β-actin were purchased from Abcam Inc. (Cambridge, MA, United States). The primary antibodies against GAPDH were purchased from Proteintech (Chicago, United States).

### Cell viability assay

Cell viability was analyzed by Cell Counting Kit-8 (CCK-8) assay (Bestbio, Shanghai, China). Generally, cells were cultured in 96-well plates at a density of 3 × 10^3^/well for 24 h. Then, cells were treated different concentrations of compounds for 72 h. Supernatant was totally removed, and 100 μL of CCK-8 solution was added to each well and cultured for another 1 h at 37°C. Cell viability was quantified by a SpectraMax M2e (Molecular Devices, San Jose, CA, United States) at 450 nm. Cell viability was calculated for each well as (OD450 treated cells/OD450 control cells) ×100%. Assays were performed on three independent experiments.

### Small interfering RNA knockdown and transfection

Small interfering RNA (siRNA) targeting RRM2 and scrambled siRNA were purchased from Heyuan Biotechnology (Shanghai, China). Cells were seeded in 6-well plates (2 × 10^5^cells/well). Cells were then transfected with the siRNA using jetPRIME (Polyplus, NY, United States) according to the manufacturer’s instructions. The sense sequences of the RRM2 and control siRNA were 5′-GGA​GUG​AUG​UCA​AGU​CCA​ATT-3′ (RRM2 siRNA-1), 5′-GGC​UCA​GCU​UGG​UCG​ACA​ATT-3′ (RRM2 siRNA-2); 5′-GCU​GAA​GUG​UUA​CCA​ACU​ATT-3′ (RRM2 siRNA-3); 5′-UUC​UCC​GAA​CGU​GUC​ACG​UTT-3′ (Scrambled siRNA).

### Viral transfection

Cells were seeded in 12-well plates (1 × 10^5^ cells/well). Then, cells were transfected with the virus using Polyplus (Polyplus, NY, United States) according to the manufacturer’s instructions.

### Colony formation assay

Cells were seeded into 6-well plates at a density of 500/well followed by 5-FU, HZ-A-018 or the combination treatment. After 72 h, the media was replaced with fresh drug-free medium and then the plate was incubated at 37°C for another 8 days until cells grew to visible colonies. After washing with PBS, cells were fixed with absolute ethyl alcohol for 15 min and stained.

### Apoptosis assay

Cells were seeded in 6-well plates (3 × 10^4^ cells/well) and cultured overnight in a 5% CO_2_ atmosphere at 37°C. After treatment with ACP-196, HZ-A-018, 5-FU or the combination for 72 h, cells were harvested and washed with PBS. Then, cells were stained with Annexin V-FITC Apoptosis Kit according to the manufacturer’s instructions and analyzed by flow cytometry (Becton Dickinson, Franklin Lakes, NJ, US). Assays were performed on three independent experiments.

### Western blot analysis

After treated with different concentrations of compounds, total proteins were extracted using RIPA lysis buffer. A total amount of 10–40 μg of proteins were subjected to 5%–12.5% SDS/PAGE and transferred to PVDF membrane (Bio-Rad, Hercules, CA, United States). The membranes were blocked with 5% non-fat milk at room temperature for 1 h, and then incubated with primary antibodies overnight at 4°C. After washing with Tris-buffered saline with Tween 20 (TBST), membranes were incubated with secondary antibodies at room temperature for another 1.5 h. The protein bands were visualized by adding ECL system WBKLS0050 (EMD Millipore, Billerica, MA, United States) and analyzed using Bio-Rad Laboratories Quantity One software (Bio-Rad). To quantify protein expression ratios, immunoblots were assessed by ImageJ software. To obtain the differential protein expression profiles, three different protein samples (control, 5-FU and the combination of 5-FU and HZ-A-018) were sent to Micrometer Biotech Co. (Hangzhou, China) for proteomic analysis.

### Immunofluorescence

HGC-27 cells (5 × 10^4^ cells/well) were cultured in glass bottom cell culture dish. After exposure to HZ-A-018, 5-fluorouracil or the combination, cells were then rinsed with PBS twice before fixation in 4% formaldehyde for 20 min at room temperature. After washing with PBS, cells were blocked by 3% BSA for 1 h and incubated with an anti-RRM2 antibody (1:300) at 4°C overnight and then with corresponding Alexa Fluro-labelled secondary antibodies at a 1:500 dilution for another 1 h at room temperature. Next, cells were incubated with DAPI (InvitrogenTM, Thermo Fisher Scientific) for 5 min, and immediately observed by confocal microscope (Leica SP8, Wetzlar, Germany).

### Tumor xenograft assay

All animal experiments were conducted according to the Institutional Animal Care and Use Committee (IACUC). Total amount of 5 × 10^6^ HGC-27 cells were suspended in 100 μL RPMI-1640 medium and injected subcutaneously into each 4-week-old female nude mice. Once the tumor volume had reached 100 mm^3^, tumor-bearing mice were randomly assigned to four groups, 5-FU (40 mg/kg), HZ-A-018 (100 mg/kg), or combined 5-FU and HZ-A-018. Both 5-FU and HZ-A-018 were dissolved in DMSO. 5-fluorouracil was administered intraperitoneally every two or 3 days, HZ-A-018 was orally administered once a day. Tumor volumes were determined using caliper measurements of tumor length (L) and width (W) according to the formula (L × W^2^)/2.

After the mice were sacrificed by cervical dislocation, tumors were dissected, measured and preserved for further experiments. Liver and kidney were harvested for H&E staining.

### Histological analysis and immunohistochemistry (IHC)

Sections (4 μm) from fresh tumor nodules embedded in 4% methanol-free formaldehyde were stained with anti-RRM2 antibody. Immunohistochemical staining of tumor sections was performed according to the manufacturer’s instructions. Images of immunohistochemistry were obtained under a light microscope.

### Statistical analysis

The results are expressed as the mean ± SD of at least three independent experiments. Differences between means were analyzed using Student's t test and were considered statistically significant when *p* < 0.05. Statistical analyses and data visualization were performed using GraphPad Prism, Version 6.01 (GraphPad Software Inc., San Diego, CA, United States).

## Results

### HZ-A-018 exhibited anti-proliferation activity in gastric cancer cells

**FIGURE 1:**
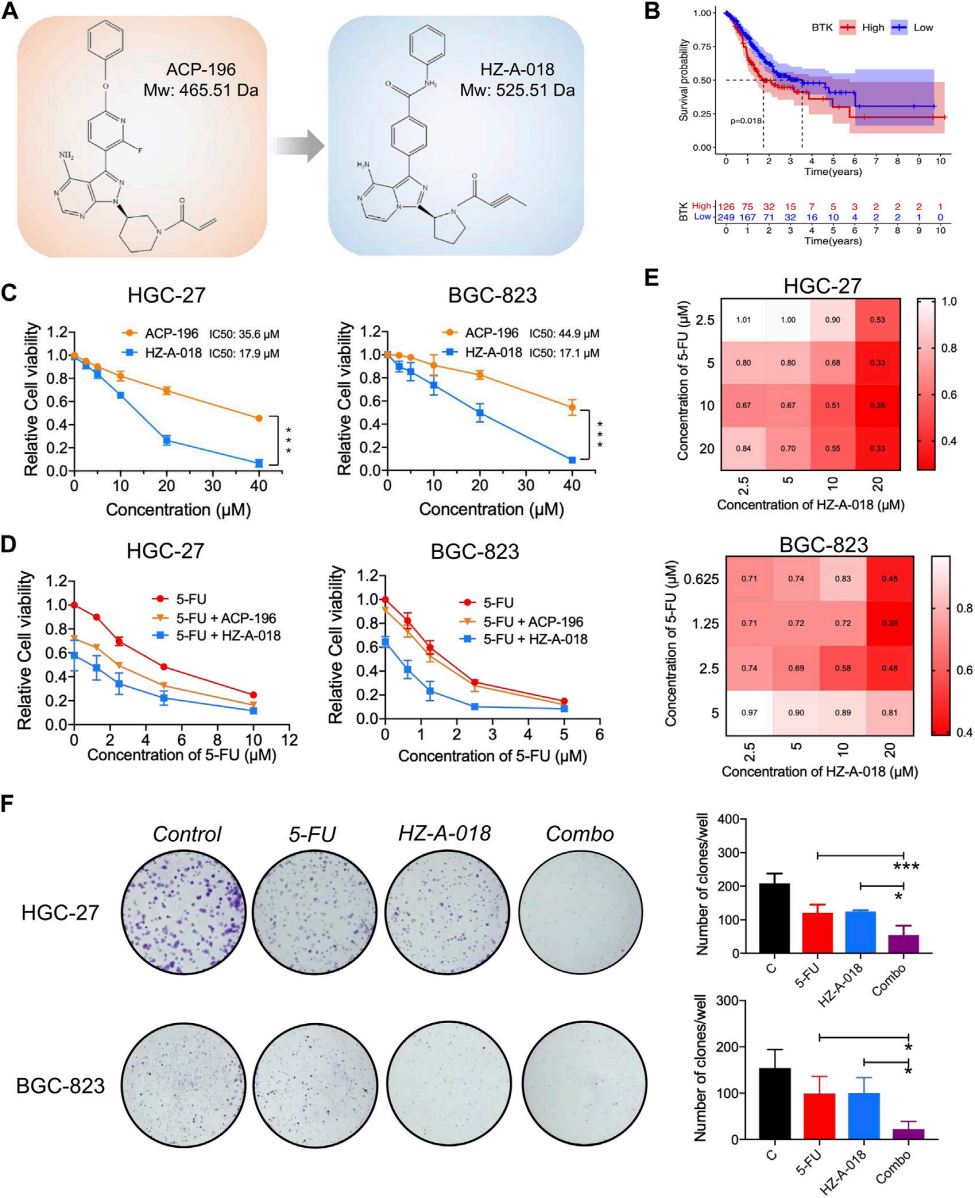
The anti-proliferative effects of HZ-A-018, ACP-196 and 5-FU in gastric cancer cells. **(A)** The chemical structure sketch of ACP-196 and HZ-A-018. **(B)** Comparison of survival between low expression and high expression of BTK. The expression data and clinical survival of gastric cancer patients were obtained from UCSC Xena (https://xenabrowser.net/). A total of 375 samples were included. **(C)** The relative cell viability after treatment with different concentrations of ACP-196 or HZ-A-018 for 72 h in HGC-27 and BGC-823 cells. **(D)** The relative cell viability after combinatorial treatment with various concentrations of 5-FU and 10 µM of ACP-196 or HZ-A-018 for 72 h in HGC-27 and BGC-823 cells. **(E)** The calculated combination index (CI) based on combinatorial treatment with 5-FU and HZ-A-018. **(F)** The inhibitory effects on colony formation after combined treatment with 5-FU (2.5 µM in HGC-27 cells and 0.25 µM in BGC-823 cells) and 10 µM of HZ-A-018. * *p* < 0.05, ** *p* < 0.01.

HZ-A-018 was a novel derivative of ACP-196 (Figure 1A), and it showed similar suppressive effects on phosphorylated BTK comparing with ACP-196 in gastric cancer cells (Supplementary Figure S1). To explore the importance of BTK in gastric cancer, database analysis was performed to show an adverse role of BTK expression level in the survival rate of gastric cancer patients (Figure 1B). Then the cytotoxicity of HZ-A-018 and ACP-196 was examined in gastric cancer HGC-27 and BGC-823 cells. After 72 h treatment, both HZ-A-018 and ACP-196 displayed dose-dependent inhibitory effects on gastric cancer cells, whereas HZ-A-018 were much more cytotoxic than ACP-196 especially at the concentration of 20 or 40 µM (Figure 1C). The IC50 values of HZ-A-018 and ACP-196 in HGC-27 and BGC-823 cells were denoted in Figure 1C. Additionally, HZ-A-018 could effectively enhance the cytotoxicity of 5-FU comparing with ACP-196 both in HGC-27 and BGC-823 cells (Figure 1D). The combination index (CI value) of HZ-A-018 and 5-FU reached down to the value of 0.3 in HGC-27 cells (Figure 1E). Additionally, the combined treatment of HZ-A-018 and 5-FU repressed the colony formation of HGC-27 and BGC-823 cells (Figure 1F). These results suggested that HZ-A-018 was more effective than ACP-196 in the treatment of gastric cancer cells.

### The combination of HZ-A-018 and 5-FU suppressed the activation of AKT/S6 pathway

**FIGURE 2:**
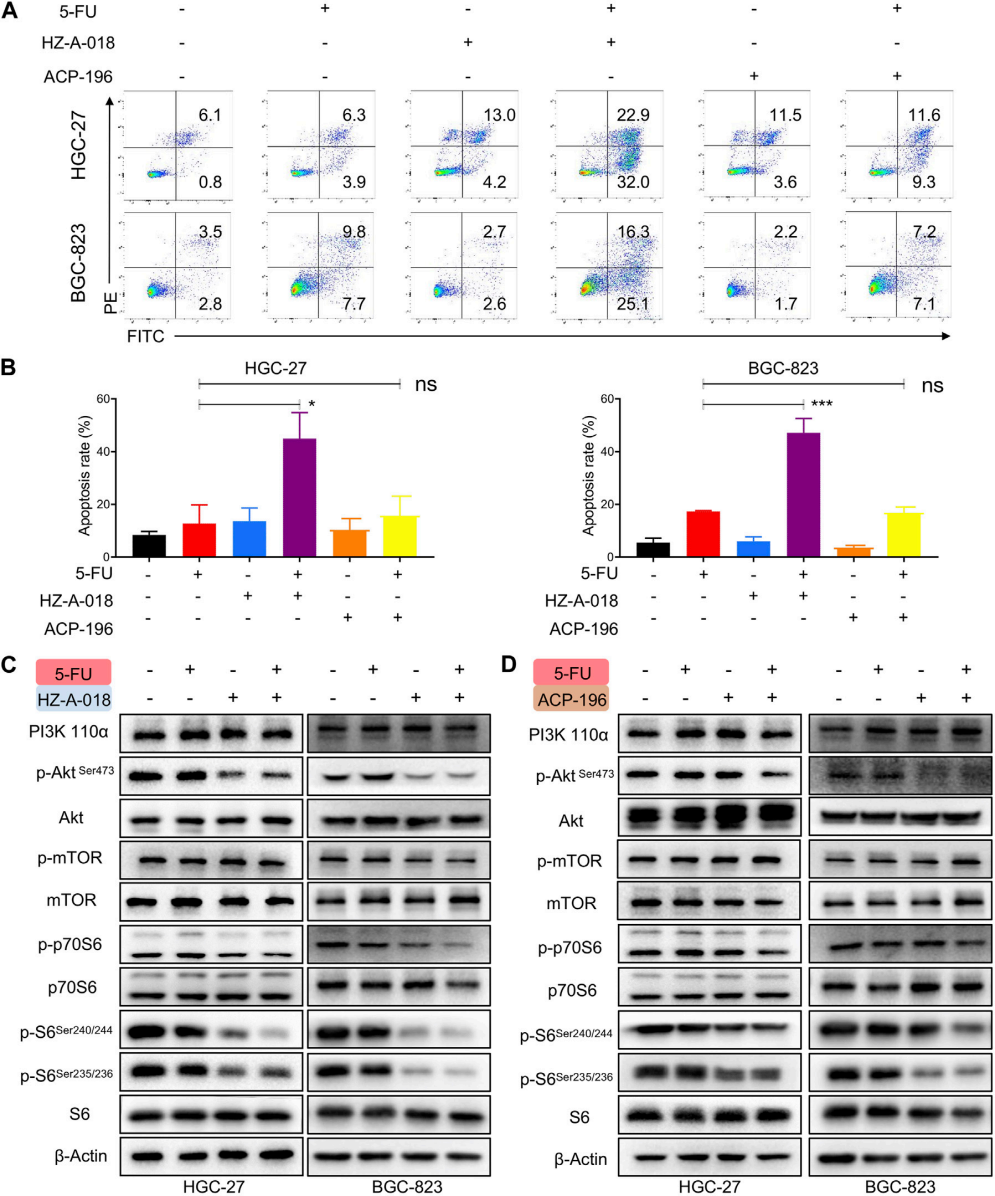
The combined effects of 5-FU and HZ-A-018/ACP-196 on apoptosis and AKT/S6 pathway of gastric cancer cells. **(A)** Flow cytometric analysis of apoptotic cells after combinatorial treatment with 5-FU (5 µM in HGC-27 cells, 0.5 µM in BGC-823 cells) and 10 µM of HZ-A-018 or ACP-196 for 72 h. **(B)** Quantitative analysis of (A). **(C)** Western blotting of AKT/S6 pathway after combinatorial treatment of 5-FU and HZ-A-018 for 24 h. **(D)** Western blotting of AKT/S6 pathway after combinatorial treatment of 5-FU and ACP-196 for 24 h ** *p* < 0.01. n.s. non-significant.

To examine the combined effects on apoptosis, cells were treated with 5-FU and HZ-A-018/ACP-196 and stained with AnnexinV/PI followed by flow cytometry analysis. The combined treatment of 5-FU and HZ-A-018 resulted in a substantial increase in apoptotic cells compared with mono-treatment with 5-FU or HZ-A-018, and the apoptosis rate reached up to 40% after 72 h treatment. Meanwhile, ACP-196 alone or in combination with 5-FU failed to exhibit synergistic effects in gastric cancer cells (Figures 2A, B). Meanwhile, the decreased expression of total PARP and pro-caspase 3 was found in cells with combinatorial treatment, accompanied by the elevated expression of cleaved-PARP and cleaved-caspase 3 (Supplementary Figure S2). Additionally, the inhibitory effects of combined treatment of 5-FU with HZ-A-018/ACP-196 on AKT/S6 pathway were analyzed by Western blotting. As a result, single treatment with HZ-A-018 was sufficient to suppress the phosphorylation of AKT and S6 in two gastric cancer cell lines (Figure 2C), while ACP-196 showed moderate inhibitory effects towards BGC-823, but not HGC-27 cells (Figure 2D). The combined treatment of HZ-A-018 and 5-FU further repressed the activation of AKT, and almost eliminated the expression of phosphorylated S6.

**FIGURE 3:**
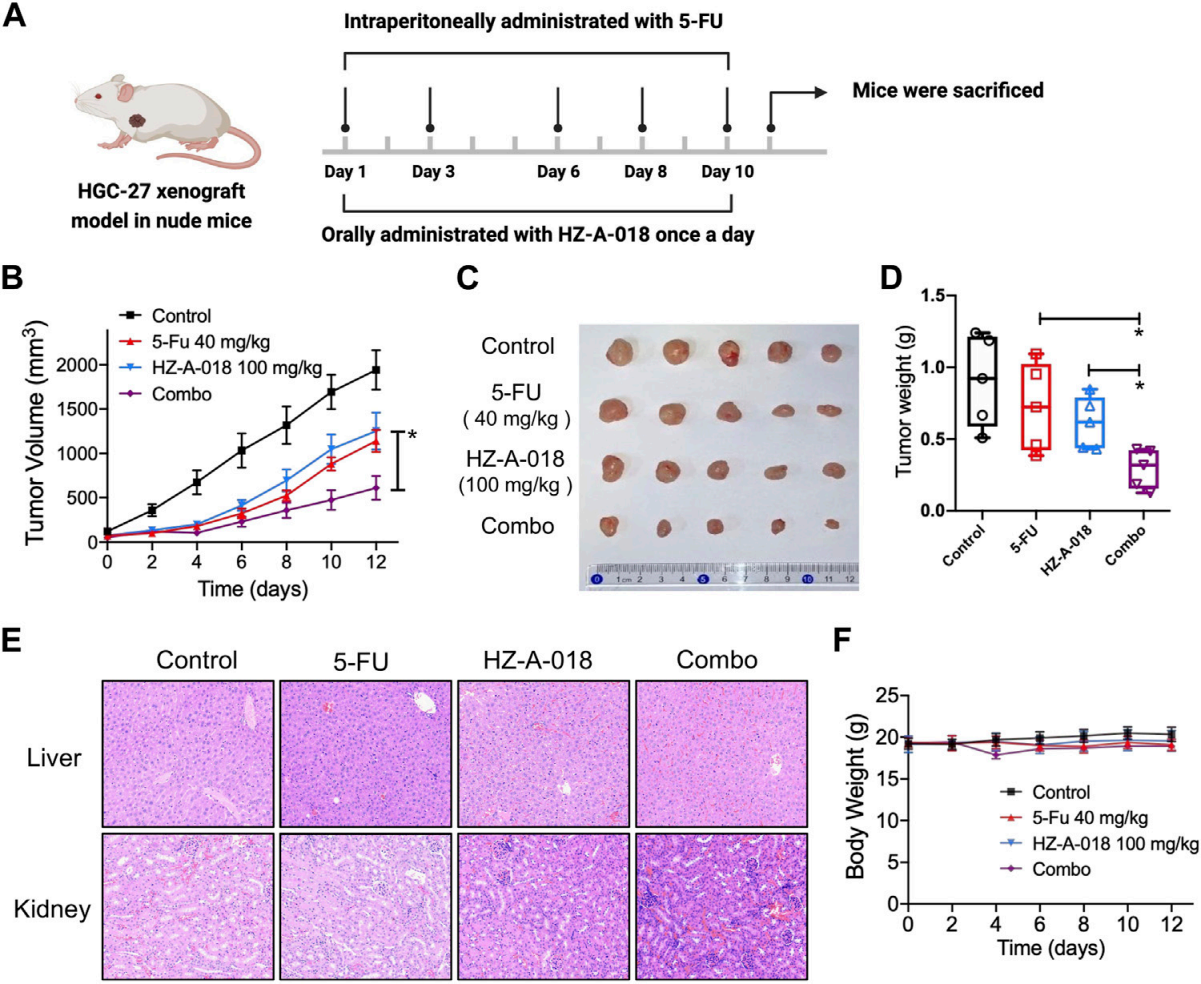
The synergistic antitumor effect of 5-FU and HZ-A-018 in HGC-27 xenograft nude mice models. **(A)** Experimental schedule of *in vivo* administration with indicated drugs. **(B)** Tumor volume was measured and recorded every other day. Data were presented as mean ± SEM. **(C)** Dissected tumor tissues in the control and treated groups. **(D)** Tumor weight of control and treated groups. **(E)** H&E stain of liver and kidney tissues in the control and treated groups. **(F)** Changes of mice body weight during experimental period. * *p* < 0.05.

In addition, the antitumor effects of combined treatment with HZ-A-018 and 5-FU were evaluated in HGC-27 xenograft models (Figure 3A). After administrated with HZ-A-018, 5-FU or the combination, tumor volume was measured and recorded every other day. Single treatment of 5-FU or HZ-A-018 could partially prevent tumor growth, and the combination of 5-FU and HZ-A-018 could achieve an approximate 70% inhibition rate (Figure 3B). Additionally, the tumor weights of dissected tumors were 0.90, 0.72, 0.61 and 0.29 g for saline, 5-FU, HZ-A-018 and combination group respectively. (Figures 3C, D). The combined treatment with HZ-A-018 and 5-FU achieved a significant smaller tumor size than single treatment. Notably, the combination treatment did not cause obvious adverse effects in liver and kidney, which was validated by H&E stain since the body weight was well maintained during the experiment (Figures 3E,F).

### The combination treatment of HZ-A-018 and 5-FU reduced RRM2 expression

**FIGURE 4:**
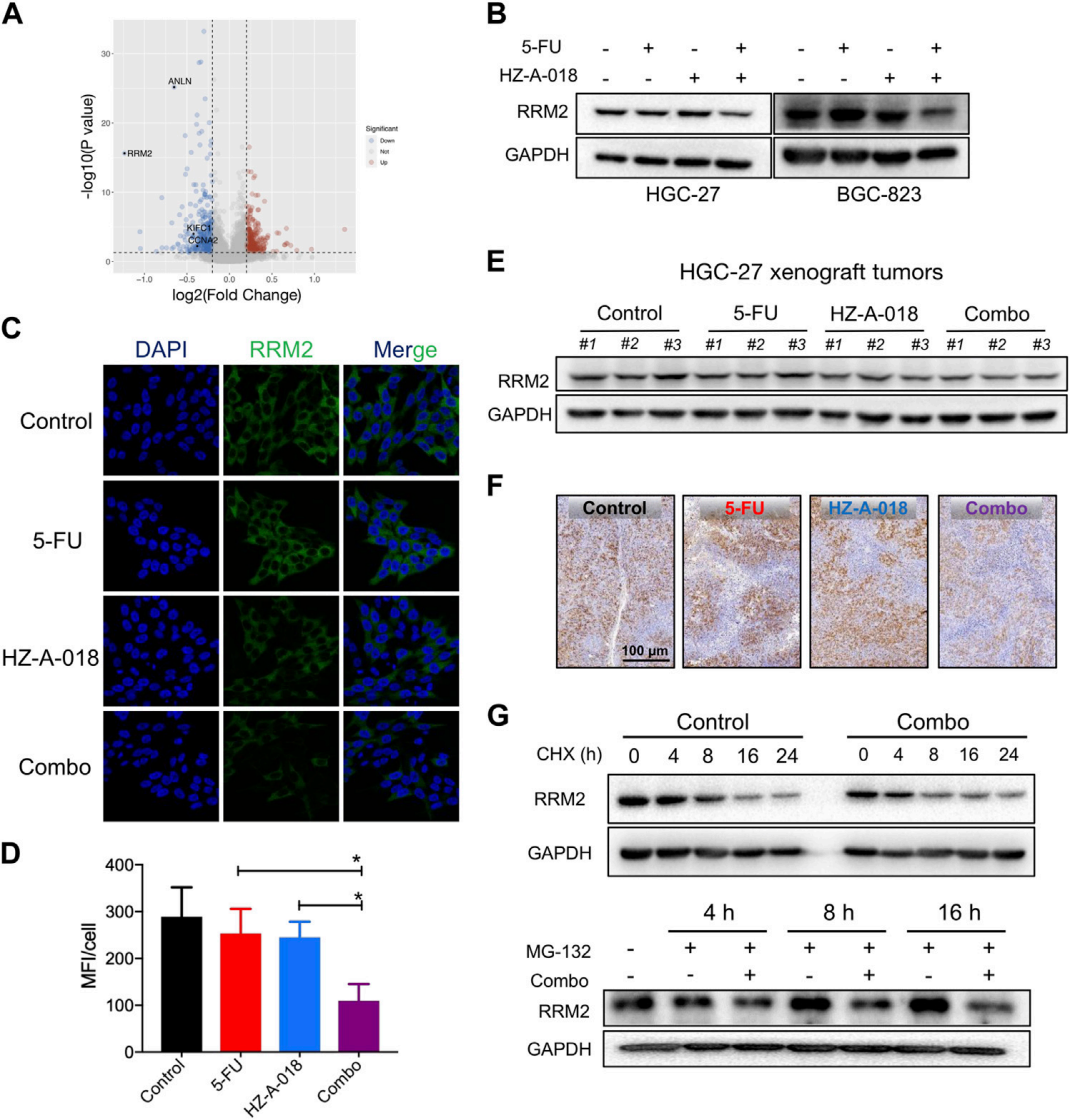
The combined treatment of 5-FU and HZ-A-018 diminished the expression of RRM2. **(A)** A Volcano plot showed the proteomics analysis comparing the combination of 5-FU and HZ-A-018 with 5-FU alone for 24 h in HGC-27 cells (*n* = 3 independent experiments). **(B)** Western blotting of RRM2 after combinatorial treatment with 5-FU and HZ-A-018 for 48 h. **(C)** Immunofluorescent images showed the decrease of RRM2 expression after combinatorial treatment for 48 h. **(D)** Quantitative analysis of (**C**). **(E)** Western blotting of RRM2 in dissected tumor tissues. **(F)** *In situ* analysis of RRM2 expression in tumor tissues by Immunohistochemistry. **(G)** The time-dependent change of RRM2 expression pretreated with 40 µM of CHX or 5 µM of MG-132. * *p* < 0.05.

Proteomic analysis was used to explore the differential protein expression of HGC-27 cells after exposure to HZ-A-018, 5-FU and the combined treatment. A significant decreased expression of RRM2 was found in combination treatment comparing with 5-FU mono-treatment, shown by the volcano plot (Figure 4A). The reduced level of RRM2 after combination treatment was validated by Western blotting (Figure 4B) and immunofluorescence in gastric cancer cells (Figures 4C, D). In animal models, combined treatment of HZ-A-018 and 5-FU also suppressed the expression of RRM2, as determined by Western blotting of tumor tissues (Figure 4E) and *in situ* immunohistochemistry of tumor sections (Figure 4F). The pre-treatment with MG-132 (a 26S proteasome inhibitor), but not cycloheximide (a protein synthesis inhibitor, CHX), attenuated the inhibitory effects of combined treatment on RRM2 protein level (Figure 4G). It was worth mentioning that, treatment with HZ-A-018 alone did not alter the expression of RRM2. Although the expression of RRM2 could be upregulated by E2F1, which contributed to the unaffected RRM2 level in mono-treatment (Supplementary Figure S3), the post-translational modification of RRM2 could not be neglected and needed to be fully investigated in future studies.

**FIGURE 5:**
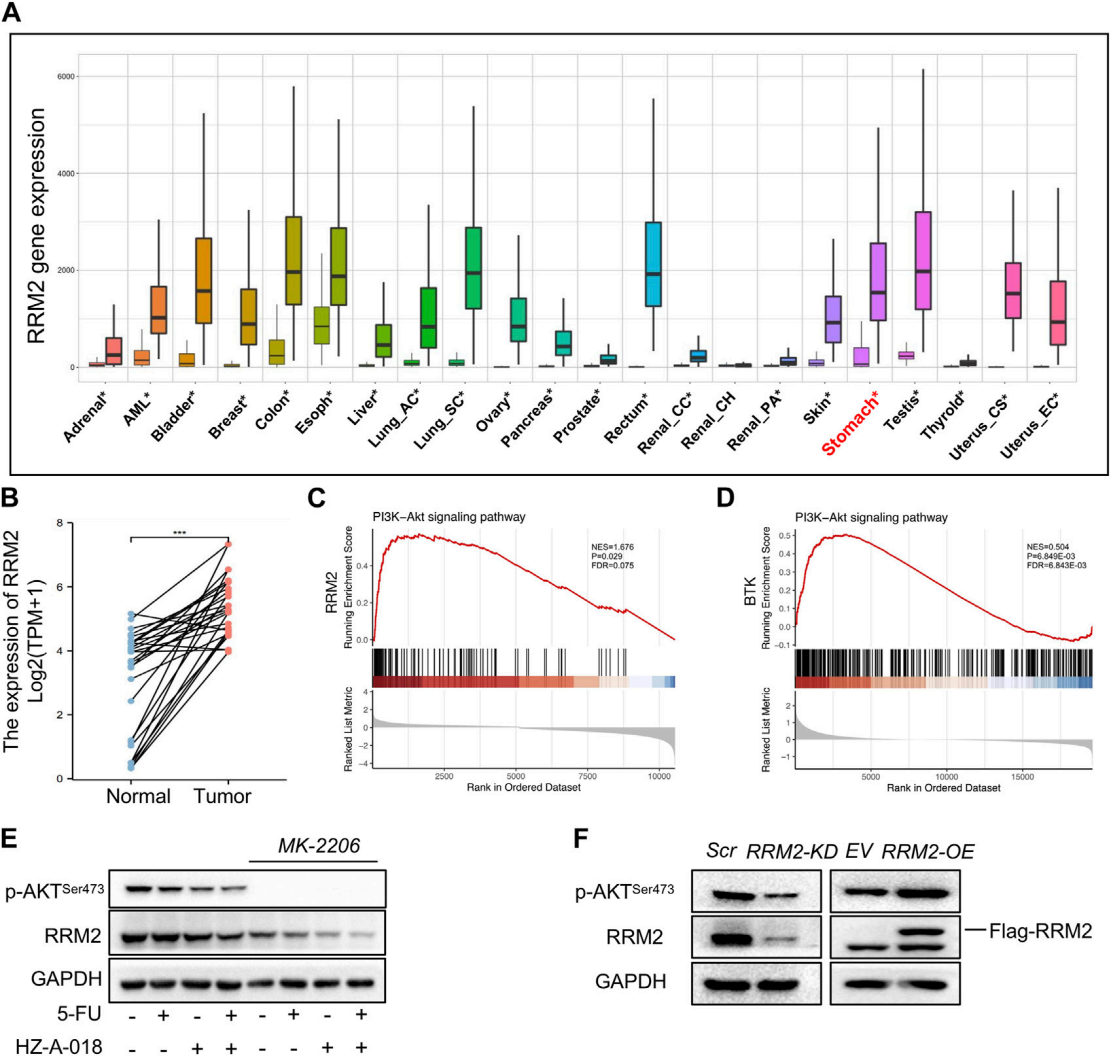
RRM2 expression in gastric cancer tissues and enrichment in PI3K pathway. **(A)** Differential expression of RRM2 in pan-cancer by RNASeq obtained from the TNMplot. Significant differences by Mann–Whitney *U* test marked with asterisk. **(B)** Paired expression of RRM2 for gastric cancer and adjacent normal tissue by RNASeq in GSE10072 dataset. **(C)** PI3K/AKT pathway enrichment plots of RRM2 obtained from GSEA. NES, normalized enrichment score of GSEA. *p* < 0.05 and FDR < 0.25 were considered statistically significant for GSEA. **(D)** PI3K/AKT pathway enrichment plots of BTK obtained from GSEA. *p* < 0.05 and FDR < 0.25 were considered statistically significant for GSEA. **(E)** The effect of MK-2206 on the expression of p-AKT and RRM2. **(F)** The effect of RRM2-OE (overexpression) and RRM2-KD (knocking down) on the expression of p-AKT and RRM2.

By analyzing the differential transcriptional expression of RRM2 in a pan-cancer panel from the TNMplot, we found an overexpression of RRM in multiple types of cancer including gastric cancer (Figure 5A), which was in consistent with the paired expression comparison of RRM2 for gastric cancer and adjacent normal tissues (Figure 5B). In addition, the enrichment plots showed a significant enrichment of RRM2 in PI3K/AKT signaling pathway (Figure 5C), and also displayed a significant enrichment of BTK in PI3K/AKT pathway (Figure 5D). We further validated the effect of AKT suppression on the activation of RRM2, and the results showed that treatment with MK-2206 could completely eliminate the expression of phosphorylated AKT, and meanwhile down-regulated RRM2 (Figure 5E). By overexpressing or knocking down RRM2 in HGC-27 cells, the expression of p-AKT seemed to be positively regulated by RRM2 (Figure 5F). Therefore, these results suggested a positive regulatory loop between RRM2 and AKT.

### RRM2 dictated the malignancy of gastric cancer cells

**FIGURE 6:**
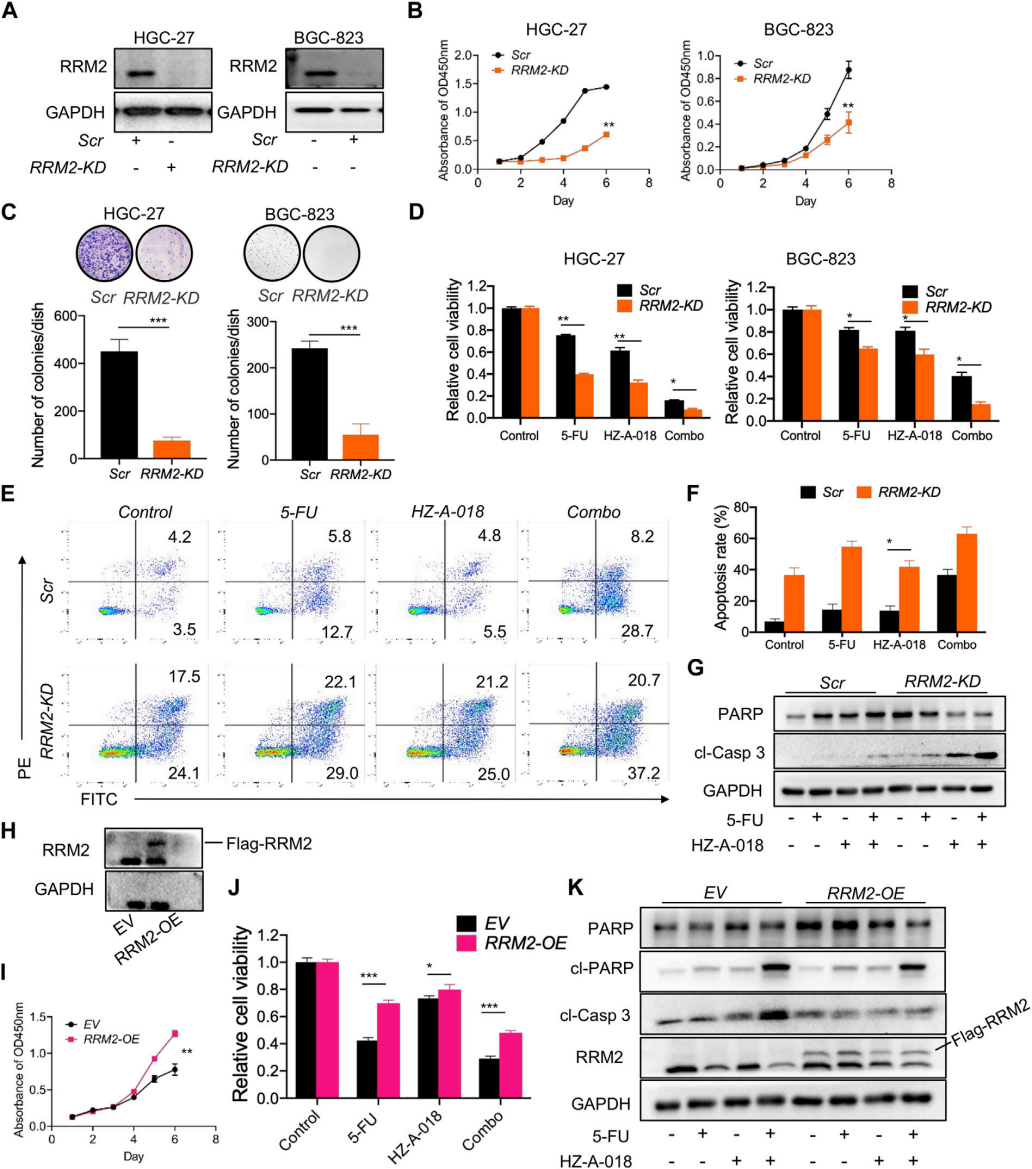
The expression of RRM2 influenced biological phenotypes of gastric cancer cells. **(A)** The successful knockdown (KD) of RRM2 by using siRNA in HGC-27 and BGC-823 cells. **(B)** RRM2-KD significantly reduced the proliferation of both HGC-27 and BGC-823 cells comparing with scrambled (Scr) control indicated by CCK-8 assay. **(C)** RRM2-KD significantly prevented the colony formation of both HGC-27 and BGC-823 cells. **(D)** RRM2-KD significantly sensitized gastric cancer cells to the combined treatment of 5-FU and HZ-A-018 indicated by CCK-8 assay. **(E)** RRM2-KD significantly induced the apoptosis after combined treatment of 5-FU and HZ-A-018 in HGC-27 cells. **(F)** Quantitative analysis of (E). **(G)** RRM2-KD augmented the expression of cleaved-caspase 3 in HGC-27 cells. **(H)** Overexpression of RRM2 (RRM2-OE) *via* viral transfection was validated by Western blot. **(I)** RRM2-OE obviously accelerated the proliferation rate of HGC-27 cells. **(J)** RRM2-OE attenuated the anti-proliferation effects of combined treatment of 5-FU and HZ-A-018 in HGC-27 cells. **(K)** Western blots of apoptotic proteins after HGC-27 cells were transfected with empty vehicle (EV) and RRM2-OE. * *p* < 0.05, ** *p* < 0.01, *** *p* < 0.001.

The role of RRM2 in the proliferation and drug sensitivity of gastric cancer cells was determined by knocking down (KD) or overexpressing (OE) RRM2 before treatment with 5-FU, HZ-A-018 or the combination (Figure 6A). As a result, RRM2-KD substantially hampered the growth rate of both HGC-27 and BGC-823 cells (Figure 6B), and dramatically diminished the number of colonies formed by HGC-27 and BGC-823 cells (Figure 6C). Importantly, RRM2-KD significantly sensitized gastric cancer cells to the treatment of 5-FU, HZ-A-018 or the combination (Figure 6D). As determined by flow cytometry, the proportion of apoptotic HGC-27 cells increased after simply knocking down of RRM2, and drug induced apoptosis was also corroborated after RRM2 knocking down (Figures 6E, F), which was further manifested by the increased expression of cleaved caspase 3 (Figure 6G).

On the other hand, the overexpression of RRM2 was established in HGC-27 cells *via* virus transfection (Figure 6H). Overexpression of RRM2 could obviously accelerate cell growth after a 6-day incubation (Figure 6I), and meanwhile conferred drug resistance to 5-FU, HZ-A-018 or the combination (Figure 6J), which was confirmed by the down-regulation of cleaved caspase 3 (Figure 6K).

## Discussion

Gastric cancer is the third leading cause of cancer-related death in the world. Currently, the most commonly used therapeutic strategy relies largely on chemotherapy, probably due to the complexity of pathological subtype in gastric cancer (Chen et al., 2020). In recent years, BTK inhibitors, for instance Ibrutinib and Acalabrutinib (ACP-196), have achieved success in the treatment of lymphocytic leukemia, whereas their clinical activity against solid tumors is limited (Overman et al., 2020), (Tempero et al., 2021). Since the expression of BTK was correlated with the prognosis of gastric cancer patients, we aimed to examine the anti-cancer activity of BTK inhibitors using gastric cancer cell lines. HZ-A-018 was a novel derivative of ACP-196, and was currently under an ongoing clinical trial (NCT04173455) to treat B Cell lymphoma. Herein, HZ-A-018 presented stronger anti-proliferation activity than ACP-196 in gastric cancer cells, and could sensitize 5-FU *via* the suppression of RRM2 both *in vitro* and *in vivo*.

To explore the underlying mechanisms, we found that HZ-A-018 was much more efficient in the suppression of AKT/S6 pathway than ACP-196, which probably contributed to the anti-cancer activity of HZ-A-018 in gastric cancer (Su et al., 2021). Meanwhile, HZ-A-018, but not ACP-196, showed synergistic effects when in combined treatment with 5-FU both *in vitro* and *in vivo*, while causing negligible adverse effects in mice (Dong et al., 2022). In breast cancer cells, the overexpression of AKT could upregulate RRM2 expression, leading to enhanced DNA repair and protection from apoptosis (Shah et al., 2014). Another studies reported the downregulation of RRM2 was found along with the downregulation of AKT pathway (Zhang et al., 2020), (Huang et al., 2019). Therefore, these studies verified the importance of AKT/S6 inhibition to exert synergistic effects with 5-FU. On the other hand, a recent study reported that RRM2 was able to stabilize ANXA1 and activate the AKT pathway (Xiong et al., 2021), suggesting a positive feedback loop between AKT and RRM2 (Chen et al., 2021b), (Rieunier et al., 2021). Herein, the combination of HZ-A-018 and 5-FU was found to downregulate RRM2 at the protein translational level, whereas the direct regulatory mechanisms of AKT/S6 on RRM2 was not illuminated and needed to be investigated in the future study.

In the treatment of gastric cancer, 5-Fu was the first line chemotherapy for decades, but the molecular determinants that decide the drug sensitivity of 5-FU is yet to be found (Sethy and Kundu, 2021). A recent study found that gene expression of RRM2 was associated with the metabolism of 5-FU and platinum (Wang et al., 2021), and the gene overexpression of RRM2 was shown particular in tumor tissues (Kidd et al., 2005). Although there was a study showed that RRM2 stimulation enhanced tumor invasiveness in gastric cancer cells (Zhong et al., 2016), the correlation between RRM2 expression and 5-FU drug sensitivity remains largely unknown. Herein, we found that overexpression of RRM2 not only accelerated cell growth, but also conferred gastric cancer cells with 5-FU resistance. Knocking down of RRM2 significantly perturbed cell growth rate and meanwhile sensitized gastric cancer cells to chemotherapy, suggesting the possibility of insufficient suppression of RRM2 by using HZ-A-018 and the existence of alternative molecular targets of HZ-A-018 in gastric cancer cells. These data enlightened the therapeutic role RRM2 in treating gastric cancer, whereas the development of RRM2-targeted inhibitor was still an unmet need.

To conclude, our work demonstrated the anti-cancer activity of HZ-A-018 in gastric cancer, and demonstrated the synergistic effects of combination treatment with HZ-A-018 and 5-FU through the inhibition of RRM2 *via* AKT/S6 suppression, thereby providing a novel therapeutic strategy in gastric cancer.

## Data Availability

The datasets presented in this study can be found in online repositories. The names of the repository/repositories and accession number(s) can be found in the article/Supplementary Material.
