# Supplementary material for: HZ-A-018, a novel inhibitor of Bruton tyrosine kinase, exerts anti-cancer activity and sensitizes 5-FU in gastric cancer cells
Source: Front Pharmacol. 2023 Mar 22;14:1142127. doi: 10.3389/fphar.2023.1142127 (PMC10073700; doi:10.3389/fphar.2023.1142127)
Supplement: Supplementary file 1 [file DataSheet1.pdf]

**SUPPLEMENTARY DATA**

**HZ-A-018, a novel inhibitor of Bruton tyrosine kinase, exerts anti-cancer activity and sensitizes 5-FU in gastric cancer cells**

Danjing Liu[1,2]*, Wei Xu[1,2]*, Bin Lin[1,3]*, Cong Ji[1,2]*, Minmin Shen[1,4], Shuyin Shen[1], Junjie Ma[1,2], Xinglu Zhou[5], Youyou Yan[2], Bo Zhang[1,2,6#], Nengming Lin[1,2,6,7#]

1 College of Pharmaceutical Sciences, Hangzhou First People's Hospital, Zhejiang Chinese Medical University, Hangzhou Zhejiang 311402, China

2 Key Laboratory of Clinical Cancer Pharmacology and Toxicology Research of Zhejiang Province,Affiliated Hangzhou First People's Hospital, Zhejiang University School of Medicine, Hangzhou Zhejiang 310006, China

3 Key Laboratory of Intelligent Pharmacy and Individualized Therapy of Huzhou, Changxing People’s Hospital, Huzhou, Zhejiang 313100, China

4 Huzhou Central Hospital, Affiliated Huzhou Hospital, Zhejiang University School of Medicine,Huzhou Zhejiang 313009,China

5 Hangzhou Hezheng Pharmaceutical Co. Ltd., Hangzhou Zhejiang 310018, China

6 Cancer center, Zhejiang University, Hangzhou Zhejiang 310058, China

7 Westlake Laboratory of Life Sciences and Biomedicine of Zhejiang Province, Hangzhou Zhejiang, 310024, China

* These authors contributed equally to this work.

**Figure S1**

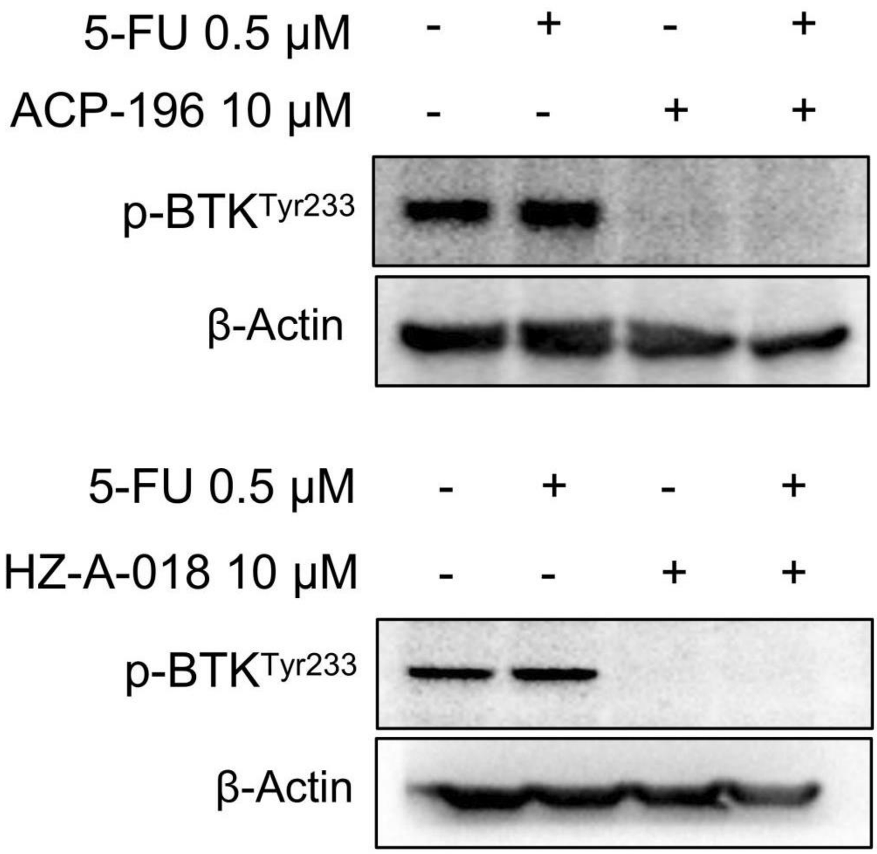


**Figure S1.** The inhibitory effects of ACP-196 and HZ-A-018 on phosphorylated BTK. HGC-27 cells were treated with 5-FU, ACP-196, HZ-A-018 or the combination as indicated for 8 h before western blotting assay.

**Figure S2**

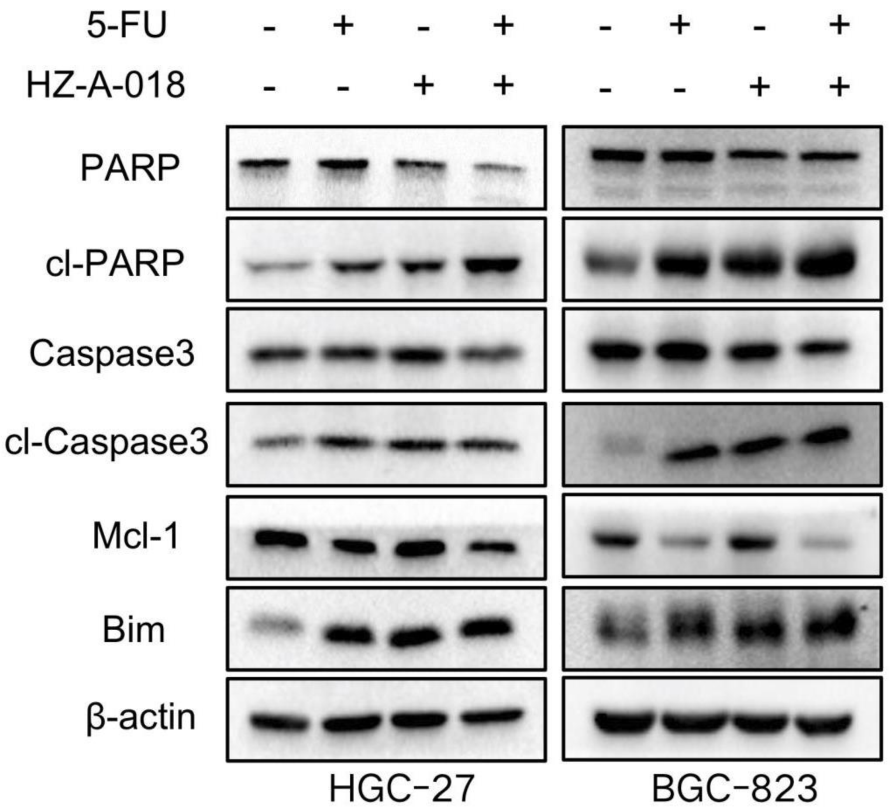


**Figure S2.**The combined effects of 5-FU and HZ-A-018 on apoptotic proteins in gastric cancer cells. After 48 h treatment with 5-FU (5 μM in HGC-27 cells, 0.5 μM in BGC-823 cells), 10 μM of HZ-A-018 or the combination, total cell lysates were collected and analyzed by western blotting.

**Figure S3**

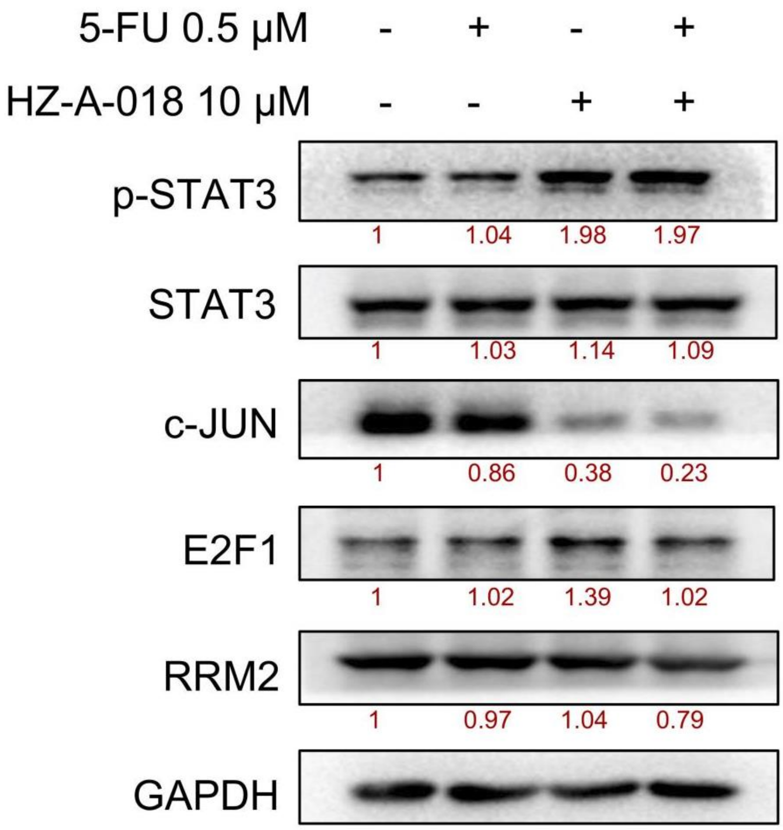


**Figure S3.** The combinatorial effects of 5-FU and HZ-A-018 on RRM2 regulating upstream proteins. HGC-27 cells were treated with 5-FU, HZ-A-018 or the combination for 24 h before western blotting assay.
